# Supplementary material for: Evidence supported by Mendelian randomization: impact on inflammatory factors in knee osteoarthritis
Source: Front Med (Lausanne). 2024 May 28;11:1382836. doi: 10.3389/fmed.2024.1382836 (PMC11165061; doi:10.3389/fmed.2024.1382836)
Supplement: Supplementary file 1 [file Data_Sheet_1.ZIP › data/GCST90274789_5e6/789.forest.pdf]

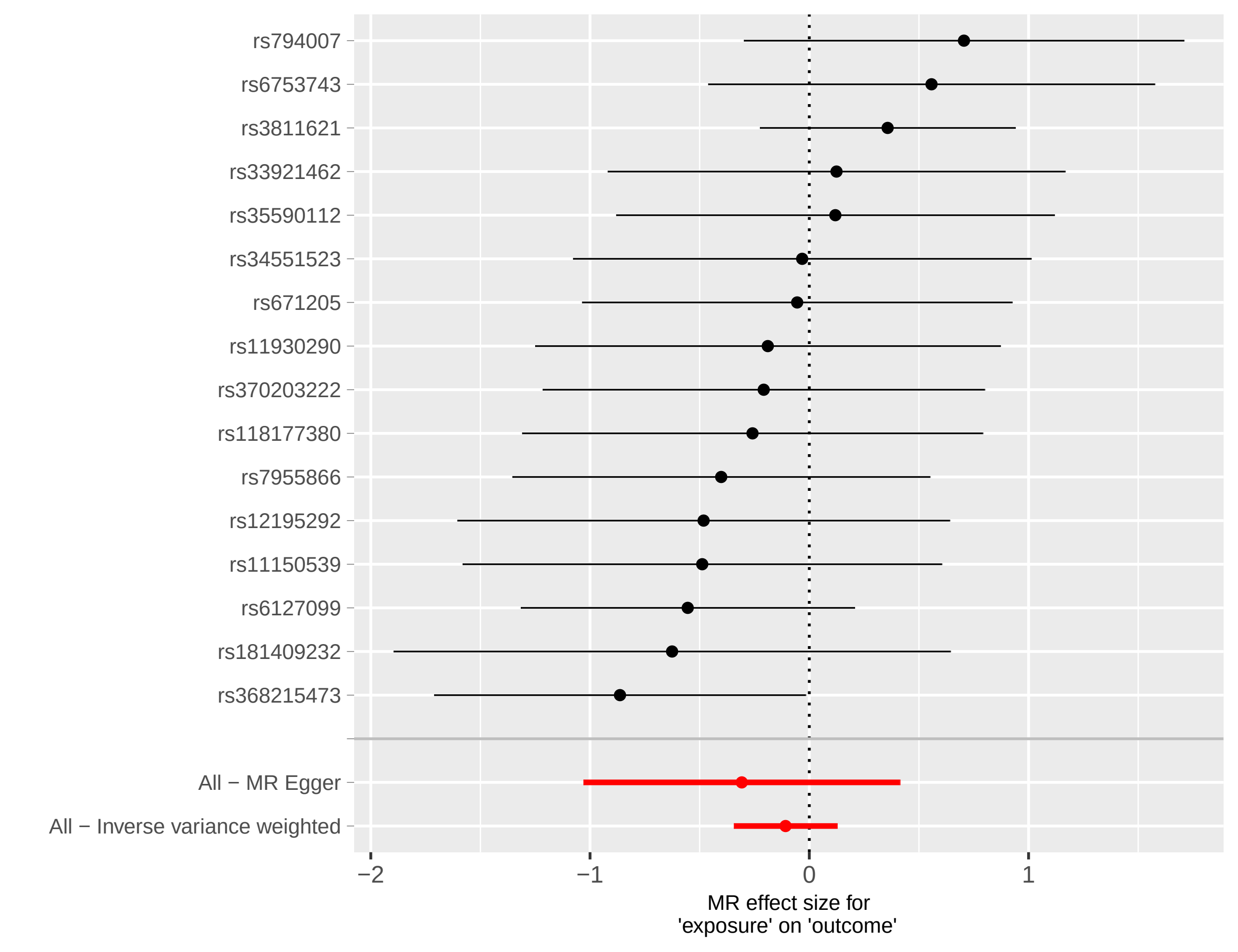
rs794007
rs6753743
rs3811621
rs33921462
rs35590112
rs34551523
rs671205
rs11930290
rs370203222
rs118177380
rs7955866
rs12195292
rs11150539
rs6127099
rs181409232
rs368215473
All − MR Egger
All − Inverse variance weighted
−2
−1
0
1
MR effect size for
'exposure' on 'outcome'
